# Effect of Adoptive Transfer or Depletion of Regulatory T Cells on Triptolide-induced Liver Injury

**DOI:** 10.3389/fphar.2016.00099

**Published:** 2016-04-22

**Authors:** Xinzhi Wang, Lixin Sun, Luyong Zhang, Zhenzhou Jiang

**Affiliations:** ^1^Jiangsu Key Laboratory of Drug Screening, Jiangsu Center for Pharmacodynamics Research and Evaluation, China Pharmaceutical UniversityNanjing, China; ^2^Key Laboratory of Drug Quality Control and Pharmacovigilance, China Pharmaceutical University, Ministry of EducationNanjing, China; ^3^Jiangsu Key Laboratory of Traditional Chinese Medicine Evaluation and Translational ResearchNanjing, China

**Keywords:** regulatory T cell (Treg), triptolide, interleukin-10, hepatotoxicity, adoptive transfer of Tregs, depletion of Tregs, suppressor of cytokine signaling (SOCS), notch signaling

## Abstract

**Objective:** The aim of this study is to clarify the role of regulatory T cell (Treg) in triptolide (TP)-induced hepatotoxicity.

**Methods:** Female C57BL/6 mice received either adoptive transfer of Tregs or depletion of Tregs, then underwent TP administration and were sacrificed 24 h after TP administration. Liver injury was determined according to alanine transaminase (ALT) and aspartate transaminase (AST) levels in serum and histopathological change in liver tissue. Hepatic frequencies of Treg cells and the mRNA expression levels of transcription factor Forkhead box P3 and retinoid orphan nuclear receptor γt (RORγt), interleukin-10 (IL-10), suppressor of cytokine signaling (SOCS), and Notch/Notch ligand were investigated.

**Results:** During TP-induced liver injury, hepatic Treg and IL-10 decreased, while T helper 17 cells cell-transcription factor RORγt, SOCS and Notch signaling increased, accompanied with liver inflammation. Adoptive transfer of Tregs ameliorated the severity of TP-induced liver injury, accompanied with increased levels of hepatic Treg and IL-10. Adoptive transfer of Tregs remarkably inhibited the expression of RORγt, SOCS3, Notch1, and Notch3. On the contrary, depletion of Treg cells in TP-administered mice resulted in a notable increase of RORγt, SOCS1, SOCS3, and Notch3, while the Treg and IL-10 of liver decreased. Consistent with the exacerbation of liver injury, higher serum levels of ALT and AST were detected in Treg-depleted mice.

**Conclusion:** These results showed that adoptive transfer or depletion of Tregs attenuated or aggravated TP-induced liver injury, suggesting that Tregs could play important roles in the progression of liver injury. SOCS proteins and Notch signaling affected Tregs, which may contribute to the pathogenesis of TP-induced hepatotoxicity.

## Introduction

Triptolide is isolated from the traditional Chinese medicine TWHF, which exhibits notable immune-regulative effects (Liu, 2011). TP has demonstrated a promising effect on the treatment of renal transplantation and autoimmune diseases in animal models by regulation of Tregs (Li et al., 2010; Zhou et al., 2011). Despite the benefits it provides, acute overdose or long-term administration of TP can cause severe liver injury and even death (Fu et al., 2011). The occurrence of drug-induced liver injury is a major problem in all phases of clinical drug development. In most cases, the mechanism of TP-induced hepatic injury remains unclear.

Several reports have showed that Tregs play an important role in liver disorders (Sennello et al., 2005; Stross et al., 2012). Treg maintains immune homeostasis by suppressing excessive immune responses which otherwise would result in serious tissue damage (Kim et al., 2007). FoxP3 is characterized as a transcription factor required for Treg development (Yagi et al., 2004). IL-10 is produced by Tregs and other cell types. IL-10 is crucial for preventing exaggerated inflammation and thus protecting the host from immune-mediated damage (Sabat et al., 2010). The liver is an organ of complex immune responses and mainly provides protection by tolerating harmless self and foreign antigens (Doherty and O’Farrelly, 2000). When tolerance is broken, activated immune cells induce liver injury and hepatic inflammation by releasing pro-inflammatory cytokines and chemokines, which determines the extent of liver injury (Wang et al., 2015). Accumulating evidence suggests that anti-inflammatory Treg cells and pro-inflammatory Th17 cells have antagonistic effects on the progression of liver injury (Li et al., 2012). Therefore, it is critical to modulate the immune homeostasis during liver injury. Although the decrease of Tregs in liver injury has been reported (Wang et al., 2014a), the involvement of Tregs in noninfectious hepatic injury, such as TP-induced liver injury, has not been investigated. The purpose of this study is to identify the involvement of Treg cells in TP-induced liver injury.

Suppressor of cytokine signaling proteins play a crucial role in preventing the cytokine responses and maintaining organ homeostasis. They are negative regulators of the JAK/STAT signaling pathway (Yoshimura et al., 2007). Eight SOCS proteins, SOCS1–SOCS7 and cytokine-inducible SH2-containing protein-1 (CIS-1) have been identified, of which SOCS1, 2, and 3 are the best characterized. Tregs are deficient in SOCS3 protein expression, which is needed for Treg to rapidly respond to cytokines to prevent unwarranted immune responses to self-antigens (Pillemer et al., 2007). The Notch signaling pathway control cell-fate decisions in T-cell development in the thymus and T-cell differentiation in the periphery (Deftos and Bevan, 2000). In mammals, there are four Notch receptors-Notch1–4 and five Notch ligands-Jagged-1 and -2 and Dll-1, -3, and -4 (Radtke et al., 2010). The Notch family contributes to induction of Treg cells and suppression of autoimmune diseases (Burghardt et al., 2014). Because SOCS and Notch signaling modulate differentiation and proliferation of T-helper (Th) cells. The second purpose of this study is to investigate the expression of SOCS and Notch/Notch ligands in TP-induced liver injury.

Our previous investigations have reported that hepatic Treg and IL-10 decreased in TP-induced liver injury (Wang et al., 2014a), yet it has been unknown about the potential mechanisms regulating Tregs in TP-induced hepatotoxicity. In the present study, the percentage and phenotypic features of Tregs in the livers of mice were examined. The expression of SOCS proteins and Notch signaling were investigated. Either adoptive transfer or depletion of Tregs was conducted to investigate Tregs’ roles in TP-induced liver injury. These results may lead to useful therapeutic approaches for TP-induced liver injury.

## Materials and Methods

### Chemicals

Triptolide (purity >98%) was a gift from the Dermatological Disease Research Institute of the Chinese Academy of Medical Sciences (Nanjing, China). TP was reconstituted in propylene glycol and stored at -20°C. Then, TP was freshly diluted to the appropriate concentrations with a 0.2% carboxymethylcellulose solution before use in the experiments.

### Animals and Treatment

Female C57BL/6 mice, 6–8 weeks of age, were purchased from the Vital River Experimental Animal Technology, Co., Ltd. (Beijing, China). All of the mice were housed under pathogen-free conditions and were provided with mouse chow and water *ad libitum*. The animals were maintained at a controlled temperature (22° ± 2°C) and photoperiod (12 h of light and 12 h of dark). The animals were acclimated to the laboratory for 1 week before the experiments. This study was approved by the Ethical Committee of China Pharmaceutical University, and Laboratory Animal Management Committee of Jiangsu Province (Approval No.: 2110748). The animal experiments were carried out in accordance with the approved guidelines.

The mice were divided randomly into five groups (*n* = 12/group): (i) vehicle-treated control group, in which mice were given 0.2% carboxymethylcellulose solution (i.g.) at 24 h prior to sacrifice; (ii) TP group, in which mice were given TP (500 μg/kg, i.g.) at 24 h before sacrifice; (iii) adoptive transfer of Treg-pretreated TP group, in which mice were given CD4^+^CD25^+^Tregs (1 × 10^6^, i.v.) at 24 h before TP administration, then underwent TP administration and sacrifice at 24 h after TP administration; (iv) adoptive transfer of Teff-pretreated TP group, in which mice were given CD4^+^CD25^-^Teffs (1 × 10^6^, i.v.) at 24 h before TP administration, then underwent TP administration and sacrifice at 24 h after TP administration; (v) anti-CD25-pretreated TP group, in which mice were given purified anti-CD25 antibodies (200 μg, i.p., clone: PC61) at 48 h before TP administration (Tenorio et al., 2010), then underwent TP administration and sacrifice at 24 h after TP administration.

### Cell Purification

Mouse CD4^+^CD25^+^Tregs and CD4^+^CD25^-^Teffs were isolated from the spleen with a MACS Treg isolation kit (Miltenyi Biotec, Auburn, CA, USA). The purity of the Treg cell was above 90%, as assessed by flow cytometry.

### Blood Chemistry Aanalysis

The blood was collected in tubes without anticoagulant to obtain serum which was analyzed for the level of ALT and AST by using an automatic clinical analyzer (7080, HITACHI Ltd, Tokyo, Japan).

### Histopathological Evaluations

Sections from the livers were removed and fixed in 10% neutral-buffered formalin. For the histopathological examination, all of the fixed organs were processed for embedding in paraffin, sectioned, and stained with H&E.

### MNC Isolation and Labeling

Murine livers were passed through a 200-gage nylon mesh, and washed with cold PBS. The cell mixture was centrifuged at 50 × *g* for 2 min. The supernatant was then centrifuged at 800 × *g* for 10 min. For hepatic MNC isolation, the cell pellets were resuspended in 40% Percoll and centrifuged at 1250 × *g* for 15 min. Then the cell pellets were treated with lysis solution to remove erythrocytes and obtain hepatic MNCs. Next, cells were blocked with anti-CD16/32 (Becton Dickinson, San Diego, CA, USA) and labeled with anti-mouse CD4 antibodies (Becton Dickinson) before permeabilization with Cytoperm/Cytofix (Becton Dickinson) according to the manufacturer’s instructions. After permeabilization, the cells were incubated with labeled antibodies that were specific for FoxP3 (Becton Dickinson). Then, the cells were centrifuged, and the pellets were washed to remove unbound antibodies. After surface and intracellular labeling, MNCs were evaluated by flow cytometry (Calibrate; Becton Dickinson, Palo Alto, CA, USA) and the data were analyzed using FlowJo version 10 software (FlowJo, Ashland, OR, USA).

### RNA Extraction and Real-time PCR

RNA was isolated from the liver sections with TRIzol reagent (Invitrogen Life Technologies, Carlsbad, CA, USA). CDNA synthesis was performed under manufacturer’s instructions by using the RevertAid First Strand cDNA Synthesis kit (Thermo Scientific, Waltham, MA, USA). Real-time PCR was performed in a 20-μL system which contained 10 μL of 1× SYBR Green Master Mix (Vazyme Biotech, Nanjing, China), 2 μL of cDNA, 6 μL of RNase/DNase-free water and 500 nM of each primer. The thermal cycler conditions included holds for 30 s at 95°C, followed by 40 cycles of 5 s at 95°C and 10 s at 60°C. A melting curve analysis was performed for each reaction with a 65–95°C ramp. The threshold cycle at which the fluorescent signal reached an arbitrarily set threshold near the middle of the log-linear phase of the amplification for each reaction was calculated, and the relative quantity of mRNA were determined. The mRNA levels were normalized against the mRNA levels of the housekeeping gene GAPDH. The primer sequences used were shown in **Table 1**.

**Table 1:** Primers for real-time RT-PCR.

Gene	Forward primer (5′–3′)	Reverse primer (5′–3′)
GAPDH	CTTTGGCATTGTGGAAGGGCTC	GCAGGGATGATGTTCTGGGCAG
FoxP3	CACCCAGGAAAGACAGCAACC	CTCGAAGACCTTCTCACAACCA
ROR-γt	TCCATATTTGACTTTTCCCACT	GATGTTCCACTCTCCTCTTCTC
IL-10	TGAAGACCCTCAGGATGCGG	AGAGCTCTGTCTAGGTCCTGG
SOCS1	GAGTGGTTGTGGAGGGTGAGA	TGGTTTGTGCAAAGATACTGGGAAT
SOCS2	CCTGGGGTTCAGTCTGTGTTAG	TGGTGATGGTTCTTTTTTCGTTTCT
SOCS3	GAGAGCGGATTCTACTGGAGCG	ACTGGATGCGTAGGTTCTTGGTC
Notch1	CCTTCGTGCTCCTGTTCTTTGTG	GGGCTCTCTCCGCTTCTTCTTG
Notch2	ACAAGTGAAGTGCAGGAGAGGGG	CAGCGGCAGGAATAGTGAGGAG
Notch3	CTCTGTGGTGATGCTGGAGATTGA	TGCTGACAAGGCTCCCAGGTAG
Notch4	GACACTCCACCTTTCATCTCTG	GGCTTCACATTCATCTATATCTTCA
Jagged1	GCTTCTCACTCAGGCATGATAAACC	CCATAGTAGTGGTCATCACAGGTCA
Jagged2	CAGGGCAAGTTCTGTGACGAGTG	CAGGCTCAGCATTGATGCAGGT
Dll-1	TATCTCCTTTCTCCTCTTTCCCTCC	CAACTTTCGGTTTCCTCTTCGTCT
Dll-3	TGAAACCTCTGGCTCCTTTGAATG	GGTCCACCCTCTTCTCACAGTTA
Dll-4	AATCAGCTTTTCAGGAACTCGGC	ATCCCTTGGGGTGTCCTCTCC

*FoxP3, Forkhead box P3.*

### Detection of Cytokines by ELISA

Serum was used to determine the concentrations of IL-10 by ELISA (RayBio, Norcross, GA, USA) in accordance with the manufacturer’s protocol.

### Statistical Analysis

The data were expressed as the mean ± SEM. The groups were evaluated using a one-way analysis of variance (ANOVA) and Turkey’s *t*-test. *P*-values <0.05 were considered to be statistically significant.

## Results

### Adoptive Transfer of Tregs Ameliorates TP-induced Liver Injury

**FIGURE 1:**
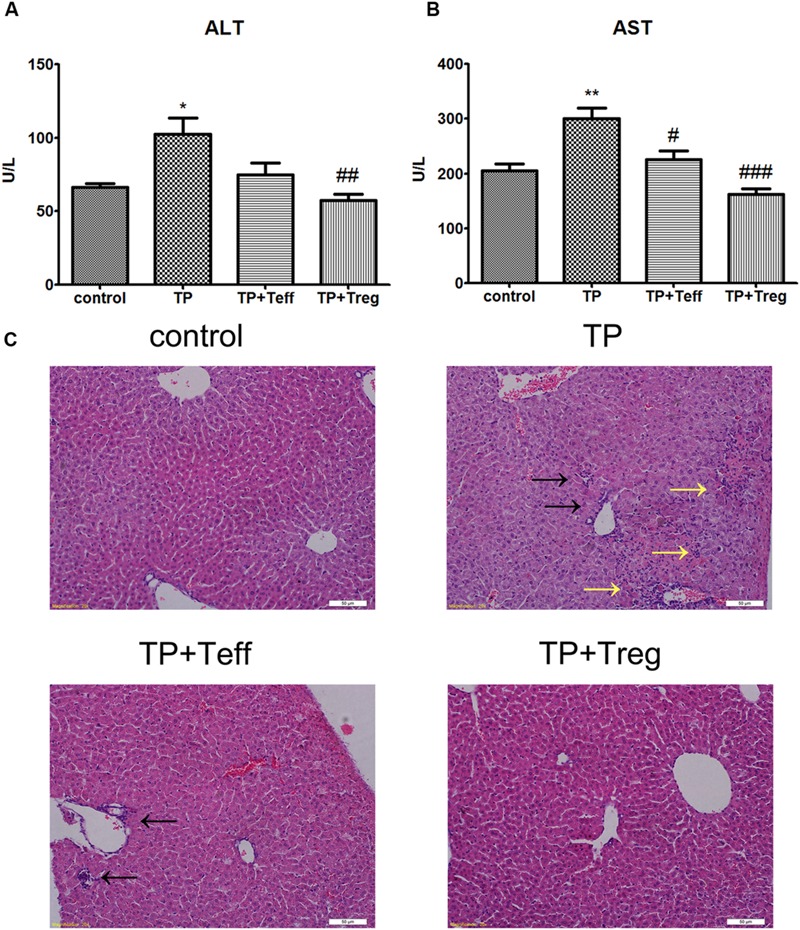
**Adoptive transfer of Tregs alleviates TP-induced liver injury.** The mice were administered with CD4^+^CD25^+^Tregs or CD4^+^CD25^-^effector T cells (1 × 10^6^; i.v.) 24 h before TP administration, then underwent TP administration (500 μg/kg; i.g.) and sacrificed 24 h after TP administration. Changes of serum ALT **(A)** and AST **(B)** levels were measured. Liver specimens were excised and fixed in 10% neutral-buffered formalin to generate tissue sections stained with H&E **(C)**. The data are shown as the mean ± SEM of six mice. ^∗^*P* < 0.05 and ^∗∗^*P* < 0.01 vs. control. ^#^*P* < 0.05, ^##^*P* < 0.01, and ^###^*P* < 0.001 vs. TP group. Black arrows shows necrosis and yellow arrows shows inflammatory cell infiltration.

The results demonstrated that ALT and AST levels significantly increased 24 hours after TP administration (**Figures 1A,B**). Histopathological changes in livers after TP administration showed necrosis with inflammatory cell infiltrating in hepatocytes (**Figure 1C**). However, adoptive transfer of Treg cells exhibited significantly lower levels of ALT and AST (**Figures 1A,B**) and ameliorated necrosis and inflammatory cell infiltrating in the liver (**Figure 1C**). Furthermore, adoptive transfer of Teffs reduced the TP-induced increase of AST, but didn’t display more obvious alleviation of TP-induced liver injury. These findings demonstrated that adoptive transfer of Treg cells attenuated TP-induced liver injury accompanied with decreasing infiltration of inflammatory cells in the liver.

### Depletion of Treg Aggravates TP-induced Liver Injury

**FIGURE 2:**
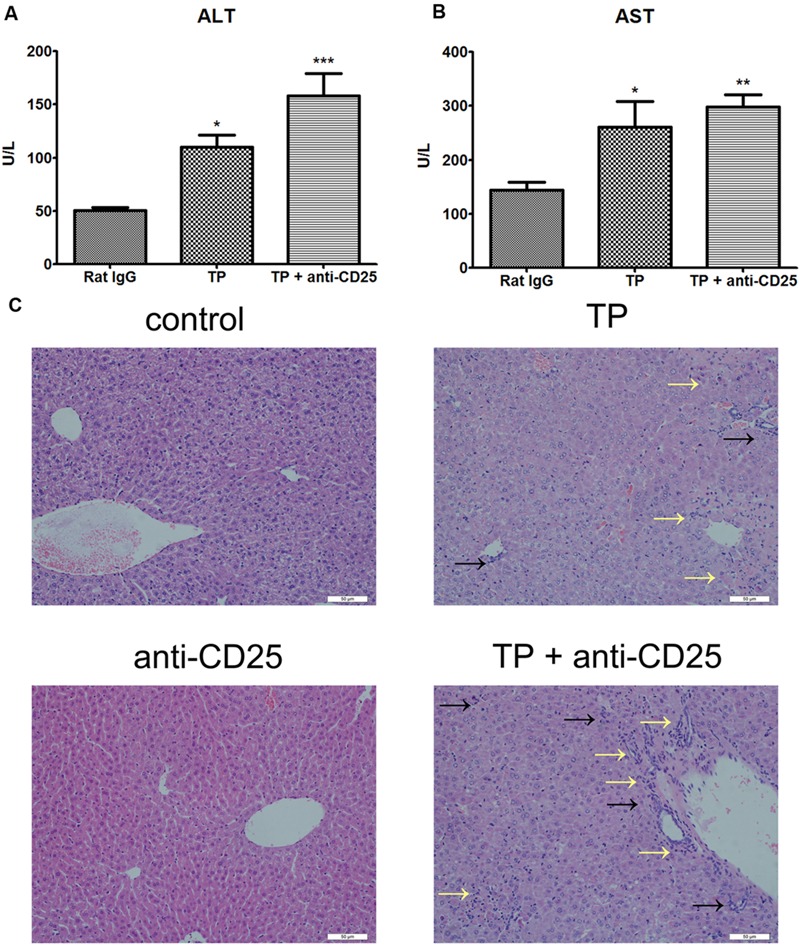
**Depletion of Tregs aggravates TP-induced liver injury.** The mice were administered with purified anti-mouse CD25 antibody (200 μg/mouse; i.p.) 48 h before the TP administration, then underwent TP administration (500 μg/kg; i.g.) and sacrificed 24 h after TP administration. Changes of serum ALT **(A)** and AST **(B)** levels were measured. Liver specimens were excised and fixed in 10% neutral-buffered formalin to generate tissue sections stained with H&E **(C)**. The data are shown as the mean ± SEM of six mice. ^∗^*P* < 0.05, ^∗∗^*P* < 0.01, and ^∗∗∗^*P* < 0.001 vs. control. Black arrows shows necrosis and yellow arrows shows inflammatory cell infiltration.

Administration of anti-CD25 antibodies and TP resulted in significantly higher ALT and AST levels in serum compared with the control group (**Figures 2A,B**). Moreover, H&E staining demonstrated severer hepatocyte necrosis and more inflammatory cell infiltration, compared with mice administered TP alone (**Figure 2C**). Mice administered the anti-CD25 antibodies alone had no obvious histopathological changes in the liver (**Figure 2C**).

### Adoptive Transfer of Tregs Modulates the Treg/Th17 Balance in Favor of Treg Cells Dominance

**FIGURE 3:**
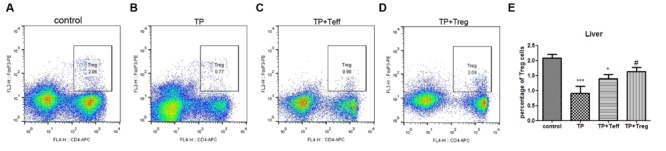
**Adoptive transfer of Tregs increases hepatic percentages of Tregs.** The mice were administered with CD4^+^CD25^+^Tregs or CD4^+^CD25^-^effector T cells (1 × 10^6^; i.v.) 24 h before TP administration, then underwent TP administration (500 μg/kg; i.g.) and sacrificed 24 h after TP administration. The percentage of CD4^+^ FoxP3^+^ T cells in the liver was detected **(A–D)** and compared **(E)**. The data are shown as the mean ± SEM of six mice. ^∗^*P* < 0.05 and ^∗∗∗^*P* < 0.001 vs. control. ^#^*P* < 0.05 vs. TP group.

**FIGURE 4:**
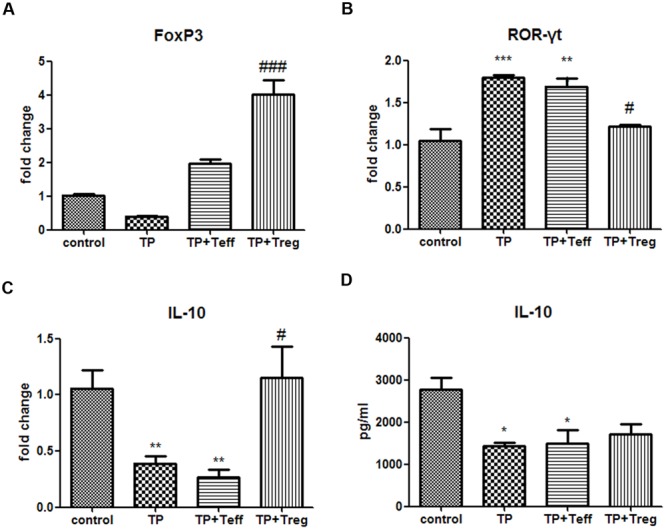
**Adoptive transfer of Tregs alters hepatic Th17/Treg balance and serum IL-10 levels.** The mice were administered with CD4^+^CD25^+^Tregs or CD4^+^CD25^-^effector T cells (1 × 10^6^; i.v.) 24 h before TP administration, then underwent TP administration (500 μg/kg; i.g.) and sacrificed 24 h after TP administration. Relative expressions of FoxP3 **(A)**, ROR-γt **(B)**, and IL-10 **(C)** were measured by real-time RT-PCR and normalized to GAPDH mRNA. The serum IL-10 level was measured by ELISA **(D)**. The data are shown as the mean ± SEM of 6 mice. ^∗^*P* < 0.05, ^∗∗^*P* < 0.01, ^∗∗∗^*P* < 0.001 vs. control. ^#^*P* < 0.05 and ^###^*P* < 0.001 vs. TP group.

Mice treated with TP had significantly lower frequencies of hepatic CD4^+^FoxP3^+^ cells (**Figures 3A,B,E**). Compared with the TP-administration group, adoptive transfer of Treg cells notably increased the frequencies of Treg cells in the liver (**Figures 3D,E**), yet adoptive transfer of Teffs didn’t alter the percentage of hepatic CD4^+^FoxP3^+^ cells (**Figures 3C,E**). Hepatic mRNA expressions of FoxP3, which is required for Treg development, showed tendencies to decrease in TP-administered mice (**Figure 4A**). Th17 cells, another subset of CD4^+^ T lymphocytes, were found to increase in TP-induced liver injury (Wang et al., 2014b). The hepatic expressions of ROR-γt, which is essential for Th17 cells differentiation, significantly increased after TP administration (**Figure 4B**). Thus, adoptive transfer of Treg not only significantly increased the hepatic mRNA expressions of FoxP3, but also remarkably reduced the mRNA levels of ROR-γt (**Figures 4A,B**). Adoptive transfer of Teffs didn’t change the mRNA expression levels induced by TP. These findings indicated that adoptive transfer of Treg contributed to regulation of the Treg/Th17 balance in favor of Treg cells dominance, which attenuated Th17 response and established a tolerance environment in TP-induced liver injury.

### Depletion of Treg Fails to Modulate the Treg/Th17 Balance and Increases Th17 Cells Expression

**FIGURE 5:**
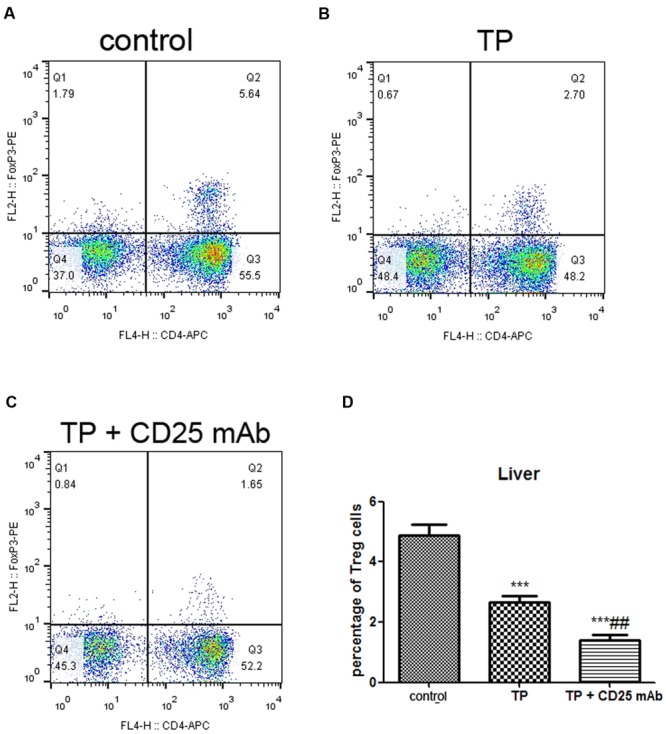
**Depletion of Tregs decreases hepatic frequencies of Treg.** The mice were administered with purified anti-mouse CD25 antibody (200 μg/mouse; i.p.) 48 h before the TP administration, then underwent TP administration (500 μg/kg; i.g.) and sacrificed 24 h after TP administration. The percentage of CD4^+^ FoxP3^+^ T cells in the liver was detected **(A–C)** and compared **(D)**. The data are shown as the mean ± SEM of six mice. ^∗∗∗^*P* < 0.001 vs. control and ^##^*P* < 0.01 vs. TP group.

**FIGURE 6:**
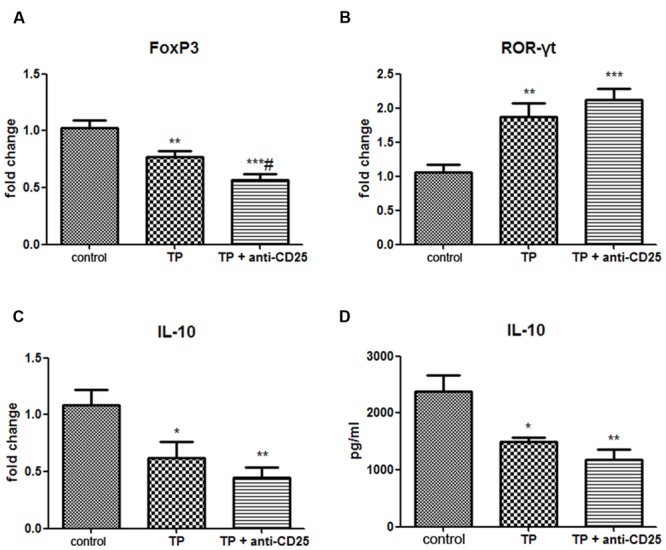
**Depletion of Tregs alters hepatic Th17/Treg balance and serum IL-10 levels.** The mice were administered with purified anti-mouse CD25 antibody (200 μg/mouse; i.p.) 48 h before the TP administration, then underwent TP administration (500 μg/kg; i.g.) and sacrificed 24 h after TP administration. Relative expressions of FoxP3 **(A)**, ROR-γt **(B)**, and IL-10 **(C)** were measured by real-time RT-PCR and normalized to GAPDH mRNA. The serum IL-10 level was measured by ELISA **(D)**. The data are shown as the mean ± SEM of six mice. ^∗^*P* < 0.05, ^∗∗^*P* < 0.01, and ^∗∗∗^*P* < 0.001 vs. control. ^#^*P* < 0.05 vs. TP group.

The percentage of Treg cells significantly decreased in the murine livers of TP and anti-CD25 co-administered mice compared with the TP-administrated mice (**Figure 5**). Hepatic mRNA expression levels of FoxP3 significantly reduced, while ROR-γt noticeably increased in TP and anti-CD25 mAb co-administered mice (**Figures 6A,B**). These results indicated that depletion of Treg resulted in failure to modulate the Treg/Th17 balance and enhancement of Th17 cell expression.

### Adoptive Transfer of Treg Cells Enhance IL-10 Secretion in Liver Tissue

Interleukin-10, mainly produced by Treg cells, plays an important role in immune tolerance and anti-inflammation (Sabat et al., 2010). The results showed the mRNA levels of IL-10 in TP-treated livers significantly decreased. Adoptive transfer of Treg significantly increased the hepatic mRNA expressions of IL-10 (**Figure 4C**), but adoptive transfer of Teffs didn’t change the mRNA expression levels of IL-10 induce by TP. The serum IL-10 levels remarkably decreased 24 h after TP administration. Tregs-pretreated mice had higher levels of IL-10, although it didn’t show significant difference (**Figure 4D**). In addition, the serum IL-10 levels in Teffs-treated mice didn’t significantly change compared with TP group. These results indicated that hepatic IL-10 contributed to Treg-mediated suppression of immune response in TP-induced liver injury.

### Depletion of Treg Diminishes IL-10 Secretion in Liver and Serum

Regulatory T-cell are likely to exhibit suppressive activity via secreting inhibitory cytokines IL-10 (Shevach, 2009). Our data showed not only hepatic mRNA expression levels of IL-10, but also serum IL-10 levels significantly reduced in TP and anti-CD25 mAb administered mice compared with the control group (**Figures 6C,D**).

### Adoptive Transfer of Tregs Down-regulates the Expressions of SOCS and Notch Signaling in TP-induced Liver Injury

**FIGURE 7:**
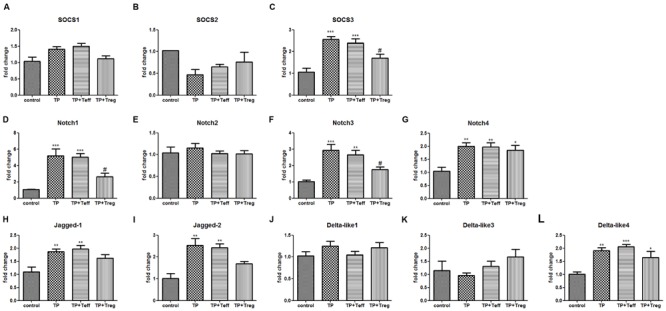
**Adoptive transfer of Tregs changes hepatic mRNA levels of SOCS signaling and Notch signaling.** The mice were administered with CD4^+^CD25^+^Tregs or CD4^+^CD25^-^effector T cells (1 × 10^6^; i.v.) 24 h before TP administration, then underwent TP administration (500 μg/kg; i.g.) and sacrificed 24 h after TP administration. Relative expressions of SOCS signaling **(A–C)** and Notch signaling **(D–L)** were measured by real-time RT-PCR and normalized to GAPDH mRNA. The data are shown as the mean ± SEM of six mice. ^∗^*P* < 0.05, ^∗∗^*P* < 0.01, and ^∗∗∗^*P* < 0.001 vs. control. ^#^*P* < 0.05 vs. TP group.

Suppressor of cytokine signaling is a negative regulator of STAT pathway and can be significantly induced under pathophysiology conditions in the liver (Yoshimura et al., 2007). The Notch signaling pathway plays critical roles in T-cell development, differentiation and liver homeostasis maintenance (Deftos and Bevan, 2000). Hepatic mRNA levels of SOCS3, Notch1, Notch3, Notch4, Jagged-1, Jagged-2, and Dll 4 significantly increased after TP administration. Adoptive transfer of Tregs remarkably inhibited the up-regulation of SOCS3, Notch1, and Notch3. Moreover, Teffs didn’t change the gene expression levels induced by TP (**Figure 7**). These results suggested that SOCS3, Notch1 and Notch 3 might affect Tregs, which might contribute to the pathogenesis of TP-induced hepatotoxicity.

### Depletion of Treg Up-regulates the Expressions of SOCS and Notch Signaling in TP-induced Liver Injury

**FIGURE 8:**
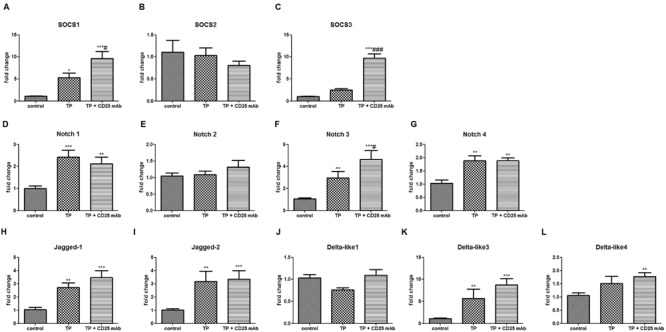
**Depletion of Tregs changes hepatic mRNA levels of SOCS signaling and Notch signaling.** The mice were administered with purified anti-mouse CD25 antibody (200 μg/mouse; i.p.) 48 h before the TP administration, then underwent TP administration (500 μg/kg; i.g.) and sacrificed 24 h after TP administration. Relative expressions of SOCS signaling **(A–C)**, Notch signaling **(D–L)** were measured by real-time RT-PCR and normalized to GAPDH mRNA. The data are shown as the mean ± SEM of six mice. ^∗^*P* < 0.05, ^∗∗^*P* < 0.01, and ^∗∗∗^*P* < 0.001 vs. control. ^#^*P* < 0.05 and ^###^*P* < 0.001 vs. TP group.

Treg depletion further increased the gene expressions of SOCS1, SOCS3, and Notch3 compared with TP-administered mice (**Figure 8**). These results suggested that SOCS1, SOCS3, and Notch 3 might modulate Tregs in the development of TP-induced hepatotoxicity.

## Discussion

Regulatory T cell can diminish potentially harmful immune responses, including inhibiting T-cell proliferation and blockading inflammatory cytokines release (Stockinger et al., 2001). Tregs are engaged in a variety of liver disorders, such as non-alcoholic fatty liver disease (Ma et al., 2007), ConA-induced liver injury (Erhardt et al., 2007) and biliary atresia (Liu et al., 2015). Tregs have reciprocal relationships with pro-inflammatory Th17 cells in the liver (Wang et al., 2014a). Th17 cell is a new lineage of CD4^+^T cell subset, which induces the immune activation and is relevant to severity of liver injury (Apostolidis et al., 2011). In the present study, adoptive transfer of Treg not only significantly increased the hepatic mRNA expressions of FoxP3, but also remarkably reduced the mRNA levels of ROR-γt. These findings indicated that adoptive transfer of Treg contributed to modulating the Treg/Th17 balance in favor of Treg cells dominance, which attenuated Th17 response and established a tolerance environment in TP-induced liver injury. In contrast, depletion of Treg noticeably decreased the hepatic mRNA levels of FoxP3 and increased the ROR-γt expressions. Moreover, depletion of Treg resulted in immune activation and aggravation of TP-induced hepatotoxicity. Thus, Treg cells played important roles in maintaining homeostasis by suppressing excessive immune response which otherwise would result in serious liver injury.

Interleukin-10 is mainly produced by Treg cells to exert effects on immune tolerance and anti-inflammation (Shevach, 2009). The mRNA levels of IL-10 in TP-treated livers significantly decreased, whereas adoptive transfer of Treg significantly increased the expressions of IL-10 in liver tissues. Moreover, depletion of Treg cell caused reduction of hepatic IL-10 expression levels and serum IL-10 levels. These observations indicated that IL-10, mainly produced and regulated by Tregs, involved in TP-induced liver injury model.

Level of certain enzyme, such as ALT and AST, in serum can be monitored as a direct indicator of hepatocyte death and liver injury. Adoptive transfer of Treg cells significantly exhibited lower levels of ALT and AST in serum and attenuated inflammatory cell infiltrating in liver tissues. However, depletion of CD25^+^ cells showed aggravation of hepatotoxicity. From these lines of evidence, explaining the immunological and biological roles of hepatic Treg cells may contribute to providing a potential strategy for managing TP-induced liver injury.

Suppressor of cytokine signaling proteins are a family of intracellular proteins that control cytokine signaling by suppressing cytokine signal transduction process (Yoshimura et al., 2007). The SOCS proteins, especially SOCS1 and SOCS3, play an essential role in mediating inflammatory responses in both immune cells and metabolic organs, such as liver (Galic et al., 2014). Conditional deletion of the SOCS3 gene revealed a role for SOCS3 as a negative regulator of the Treg subset with increased IL-10 and TGFβ production (Fitzgerald et al., 2009). In the present study, SOCS3 expression significantly increased in the liver after TP administration, which may be associated with hepatic Treg reduction. Whereas adoptive transfer of Tregs remarkably inhibited the expression of SOCS3, which may contribute to Treg expansion in the liver. In addition, depletion of Treg cells further increased the mRNA levels of SOCS1 and SOCS3 accompanied with more hepatic Treg reduction. The findings indicate that SOCS proteins are target genes for immune suppression induced by Treg cells.

The Notch signaling pathway is engaged in T cell development and differentiation (Deftos and Bevan, 2000). In the liver, the Notch pathway is indispensable for liver homeostasis (Morell et al., 2013). FoxP3 has been demonstrated to be a downstream target of Notch signaling in human cells and Notch1 signaling is involved in Treg cell differentiation (Del Papa et al., 2013). TP administration induced significant increase of hepatic mRNA levels of Notch1, Notch3, Notch4, Jagged-1, Jagged-2, and Dll 4. Adoptive transfer of Tregs significantly inhibited the up-regulation of Notch1 and Notch3, whereas Treg deficiency further increased the gene expressions of Notch3. Overall, these results revealed that Notch signaling contributed to the changes of adoptive transfer or depletion Treg in TP-induced liver injury.

In summary, the present study clearly demonstrated a potent protective role of Tregs in TP-induced liver injury and this protection may be mediated by enhancing the secretion of IL-10. In addition, SOCS proteins and Notch signaling contributed to the changes of adoptive transfer or depletion of Treg in TP-induced liver injury. These findings may place Treg cells as potential targets for pharmacotherapy, though the detailed investigations on mechanism need to be carried out in the future.

## Author Contributions

XW, LZ, and ZJ designed the experiments. XW performed the experiments. XW and ZJ analyzed and discussed the data. XW, LS, LZ, and ZJ wrote the paper. All authors contributed to the editing of the paper and to scientific discussions.

## Conflict of Interest Statement

The authors declare that the research was conducted in the absence of any commercial or financial relationships that could be construed as a potential conflict of interest.

## Abbreviations

ALT: alanine transaminase
AST: aspartate transaminase
Dll: delta-like
ELISA: enzyme linked immunosorbent assay
FoxP3: Forkhead box P3
H&E: hematoxylin and eosin
IL-10: interleukin-10
MNC: mononuclear cell
RORγt: retinoid orphan nuclear receptor γt
SOCS: suppressor of cytokine signaling
STAT: signal transducers and activators of transcription
Th17 cells: T helper 17 cells
TP: triptolide
Treg cells: regulatory T cells
TWHF: *Tripterygium wilfordii* Hook F
